# Prenatal diagnosis of cardiac rhabdomyoma: implications for predicting tuberous sclerosis complex and guiding perinatal management

**DOI:** 10.1002/uog.70240

**Published:** 2026-06-06

**Authors:** X. Cai, J. Liu, W. Zheng, Y. Jiao, Y. Lou, M. Deng, W. Zhao, K. Yan, L. Sun

**Affiliations:** ^1^ Department of Ultrasound Women's Hospital School of Medicine Zhejiang University Hangzhou China; ^2^ Institute of Medical Genetics and Development, Key Laboratory of Reproductive Genetics (Ministry of Education) and Women's Hospital School of Medicine Zhejiang University Hangzhou China; ^3^ Department of Radiology Women's Hospital School of Medicine Zhejiang University Hangzhou China; ^4^ Department of Pathology Women's Hospital School of Medicine Zhejiang University Hangzhou China; ^5^ Department of Reproductive Genetics Women's Hospital School of Medicine Zhejiang University Hangzhou China; ^6^ Department of Department of Information Technology Women's Hospital School of Medicine Zhejiang University Hangzhou China

**Keywords:** cardiac rhabdomyoma, fetal magnetic resonance imaging, genetic testing, genotype–phenotype association, pregnancy outcome, prenatal diagnosis, trio whole‐exome sequencing, tuberous sclerosis complex

## Abstract

**Objective:**

To identify prenatal predictors of tuberous sclerosis complex (TSC) in fetuses with one or more cardiac rhabdomyomas (CR), evaluate an integrated multimodal diagnostic workflow using fetal magnetic resonance imaging (MRI) and trio whole‐exome sequencing (trio‐WES) and characterize perinatal outcomes.

**Methods:**

This was a retrospective cohort study of 80 fetuses that were diagnosed prenatally with one or more CR between February 2016 and December 2024 at a single tertiary center and that received a definitive TSC diagnosis either prenatally (via genomic or clinical criteria) or by postnatal pathological verification. All fetuses underwent routine high‐resolution echocardiography. Those in which a CR was suspected were then offered the option of further investigation via fetal brain MRI and trio‐WES, to identify pathogenic variants in *TSC1*/*TSC2* genes. A definitive prenatal TSC diagnosis was assigned based on genetic testing or the presence of two major clinical features. Clinical, imaging, genetic and perinatal data were retrieved and compared between the TSC‐positive and TSC‐negative groups. Postnatal or postmortem verification was only required in cases that lacked a definitive prenatal diagnosis. Performance metrics, including sensitivity, specificity and positive (PPV) and negative (NPV) predictive values, were calculated to evaluate the diagnostic utility of tumor multiplicity and fetal brain MRI in prediction of TSC.

**Results:**

Forty‐eight (60.0%) fetuses were diagnosed with TSC and 32 (40.0%) were TSC‐negative. Tumor multiplicity had a sensitivity of 87.5% (95% CI, 74.8–95.3%) but low specificity (50.0%; 95% CI, 31.9–68.1%) for the prediction of TSC. Seventy fetuses underwent brain MRI. Among these, MRI achieved a specificity of 100.0% (95% CI, 89.1–100.0%) and identified central nervous system lesions in 89.4% of those with TSC that underwent the MRI examination, of which 81.0% were occult on ultrasound examination. Trio‐WES was performed in 31 cases. Among these, pathogenic variants were identified in 20 cases, with 85.0% of these affecting the *TSC2* gene. Trio‐WES identified pathogenic variants in five TSC‐positive cases that presented with normal fetal brain MRI, demonstrating its essential additive value for fetuses with occult imaging phenotypes, while MRI detected structural lesions in four trio‐WES‐negative cases. *TSC2* variants were associated with more severe phenotypes, including subependymal giant cell astrocytoma, whereas *TSC1* variants typically presented with milder manifestations. Sixty‐two fetuses underwent termination of pregnancy (TOP); the TOP rate was 87.5% in the TSC‐positive group and 62.5% in the TSC‐negative group. Eighteen fetuses were liveborn (six TSC‐positive and 12 TSC‐negative). The TSC‐positive infants had significantly lower birth weight compared with the TSC‐negative infants (median, 3050 g *vs* 3500 g; *P* = 0.025), despite the two groups having a similar gestational age at delivery. All liveborn TSC‐positive infants developed postnatal epilepsy.

**Conclusion:**

Multiple CR at screening ultrasound examination and a major lesion at fetal brain MRI are key prenatal predictors of TSC, but accurate diagnosis requires not only MRI but also trio‐WES, to overcome the developmental limitations imposed by the late appearance of some fetal anomalies associated with TSC and genetic limitations inherent in standard molecular analysis. Given the severe perinatal outcomes associated with TSC observed in this study, including high termination rates and postnatal epilepsy in all liveborn infants, definitive prenatal diagnosis is essential for accurate risk stratification, parental counseling and proactive neurological management. © 2026 The Author(s). *Ultrasound in Obstetrics & Gynecology* published by John Wiley & Sons Ltd on behalf of International Society of Ultrasound in Obstetrics and Gynecology.

## INTRODUCTION

Tuberous sclerosis complex (TSC) is a multisystem, autosomal‐dominant neurocutaneous syndrome caused by pathogenic variants in either the *TSC1* or the *TSC2* gene^1^, ^2^. These variants lead to hyperactivation of the mammalian target of rapamycin (mTOR) pathway, resulting in hamartomatous lesions across various vital organs^3^. Cardiac rhabdomyomas (CR) are the most common primary cardiac tumor in fetal and pediatric populations^4^. Identified as hyperechogenic masses on routine fetal echocardiography (FE), they serve as critical sentinel markers for prenatal TSC diagnosis, with an associated risk of 50–90%^4^, ^5^. Although CR often regress spontaneously postnatally, there may be long‐term consequences. The prognosis of affected fetuses is determined primarily by the severity of central nervous system (CNS) manifestations, which include intractable epilepsy and neurodevelopmental impairment^6^.

In recent years, fetal brain magnetic resonance imaging (MRI) has become established as an expert‐level diagnostic tool capable of identifying major CNS criteria, such as subependymal nodules (SEN) and cortical tubers, which are often occult on routine screening ultrasound examination^7^. The integration of trio whole‐exome sequencing (trio‐WES) further enhances diagnostic yield and assessment of recurrence risk^8^, ^9^, ^10^. Yet, despite these advances, clinicians frequently encounter diagnostic discordance. Pathogenic variants, particularly *TSC1* mutations, may present with normal initial neuroimaging due to delayed hamartoma formation or milder fetal phenotypes^6^, ^11^. Moreover, approximately 10–15% of clinically definite cases remain molecularly negative through standard sequencing due to low‐level somatic mosaicism or intronic variants^9^, ^12^, ^13^, ^14^. This diagnostic uncertainty complicates parental counseling and the planning of proactive perinatal care.

In this study, we analyzed a longitudinal cohort of 80 fetuses with prenatally diagnosed CR. While the presence of any CR serves as a major clinical criterion, we specifically aimed to investigate the predictive value of tumor multiplicity, distinguishing between single and multiple CR, and its association with a fetus's trajectory through the prenatal diagnostic workflow. We specifically examined the mechanisms underlying imaging–genomic discordance and characterized the factors influencing pregnancy outcomes and postnatal neurological trajectories. Our objective was to identify significant predictors of TSC in fetuses with one or more CR, evaluate the performance of a multimodal integrated diagnostic workflow and characterize perinatal outcomes, to refine the diagnostic framework, ensure accurate risk stratification and thus improve perinatal management of this complex population.

## METHODS

### Study design and population

This retrospective cohort study was conducted at a single tertiary referral center (Women's Hospital, School of Medicine Zhejiang University, Hangzhou, China). We reviewed the medical records of consecutive patients who underwent routine FE between February 2016 and December 2024. Patients were eligible for inclusion if FE identified one or more CR. Following the initial ultrasound diagnosis, all patients were referred routinely for a comprehensive diagnostic workup, which included the offer of fetal brain MRI, performed within 1 week after the FE examination, and trio‐WES on amniotic fluid or cord blood. Cases were excluded if incomplete clinical data or loss to follow‐up precluded definitive diagnosis of TSC.

### Diagnostic criteria and categorization

To ensure diagnostic rigor, we adapted for prenatal application the diagnostic criteria of the 2021 updated International Tuberous Sclerosis Complex Consensus Conference^1^, ^15^. The cohort was stratified into two primary groups: TSC‐positive and TSC‐negative. The prenatal diagnostic pathway followed a sequential clinical workflow: (1) initial detection of CR via screening FE; (2) subsequent fetal brain MRI, to evaluate for concurrent CNS lesions; and (3) trio‐WES to identify pathogenic variants. A diagnosis of definitive prenatal TSC (TSC‐positive) was assigned if the fetus met at least one of the following two criteria: (1) clinical criterion: presence of at least two major clinical features, which in this cohort specifically required the combination of: (a) one or more CR on ultrasound examination, and (b) at least one major brain lesion (e.g. SEN, cortical tuber or subependymal giant cell astrocytoma (SEGA)), identified via fetal MRI or autopsy; (2) genetic criterion: identification via trio‐WES of a pathogenic or likely pathogenic variant in the *TSC1* or *TSC2* gene. Conversely, TSC‐negative status was assigned to fetuses that did not meet either of the two (clinical or genetic) criteria, confirmed by benign clinical follow‐up lacking TSC manifestations if genetic testing or MRI was not performed. For cases lacking a definitive prenatal diagnosis (i.e. single CR and negative MRI and trio‐WES), postnatal follow‐up or postmortem examination provided final verification. An asymptomatic course with tumor regression confirmed TSC‐negative status, whereas the development of neurological symptoms reclassified cases as TSC‐positive. Cases without sufficient follow‐up to confirm either outcome were excluded.

### Data collection and multimodal assessment

Clinical and imaging data were extracted retrospectively from electronic medical records held in the hospital's database. FE was performed using high‐resolution ultrasound systems (Samsung HERA W10 or WS80A, Samsung Medison, Seoul, South Korea or Voluson E10 or E8, GE Healthcare, Zipf, Austria). CR were characterized by number (single *vs* multiple), maximum diameter and anatomical location. Fetal brain MRI was conducted using a 1.5‐Tesla scanner (GE Signa HDxt, GE Healthcare, Milwaukee, WI, USA) to assess specifically TSC‐associated lesions, including cortical tubers or SEN and SEGA, by their pathognomonic signal characteristics (i.e. hypointense on T2‐weighted MRI or hyperintense on T1‐weighted MRI).

Trio‐WES was performed on fetal samples obtained via amniocentesis or cordocentesis. Identified variants were classified according to the American College of Medical Genetics and Genomics (ACMG) guidelines^16^. Pregnancy outcome (live birth or termination of pregnancy (TOP)) was recorded. For liveborn infants, postnatal follow‐up data were collected, including repeat neuroimaging and the development of epilepsy. In a subset of five cases that underwent TOP, histopathological confirmation of CR was sought through fetopsy via the identification of spider cells, of which only three cases were confirmed as TSC‐positive.

### Ethical approval

This study was conducted in accordance with the principles of the Declaration of Helsinki. The study protocol was approved by the institutional review board of the Women's Hospital, School of Medicine Zhejiang University (IRB‐20250364‐R). Due to the retrospective nature of the study and the use of anonymized data, the requirement for informed consent was waived.

### Statistical analysis

Statistical analysis was performed using R software (version 4.5.2; R Foundation for Statistical Computing, Vienna, Austria) and RStudio (Posit PBC, Boston, MA, USA). Continuous variables are presented as median with interquartile range and were compared using the Wilcoxon rank‐sum test. Categorical variables are expressed as *n* (%) and were compared using Fisher's exact test or the chi‐square test. Predictive performance metrics with 95% CI, including sensitivity, specificity, positive predictive value (PPV) and negative predictive value (NPV), were calculated using the R package caret to evaluate the diagnostic utility of tumor multiplicity and MRI findings. These specific imaging characteristics were selected for evaluation because they represent the sequential, non‐invasive clinical screening steps available before invasive genetic testing. Trio‐WES was not evaluated using these metrics to avoid incorporation bias, as positive molecular findings served as a reference standard for defining the TSC‐positive cohort, and because trio‐WES was not performed uniformly across the study population. A two‐sided *P*‐value of < 0.05 was considered statistically significant.

## RESULTS

### Cohort characteristics and performance of imaging predictors

**Figure 1:**
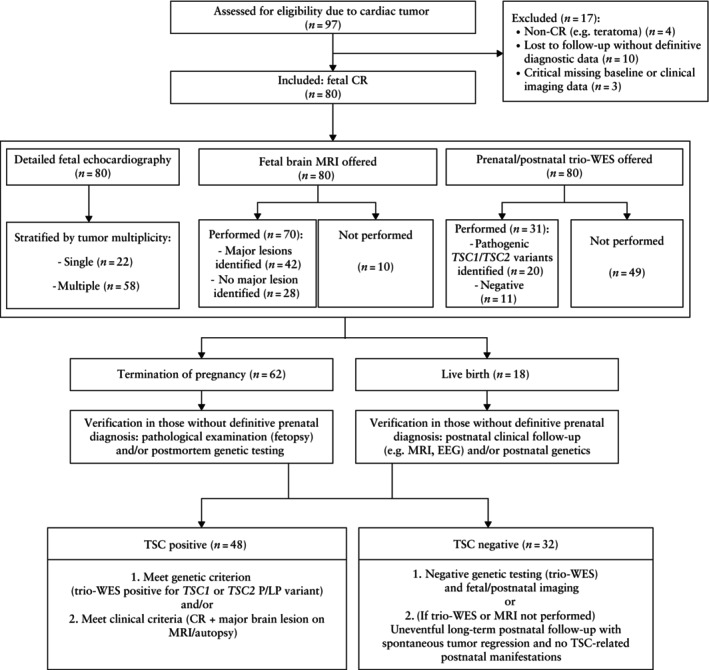
Flowchart summarizing patient recruitment, multimodal diagnostic workup and clinical categorization of the fetal cardiac rhabdomyoma (CR) cohort, showing inclusion/exclusion criteria and the definitive diagnostic pathway of tuberous sclerosis complex (TSC) (genetic *vs* clinical criteria) for this longitudinal analysis. EEG, electroencephalogram; MRI, magnetic resonance imaging; P/LP, pathogenic or likely pathogenic; trio‐WES, trio whole‐exome sequencing.

**Figure 2:**
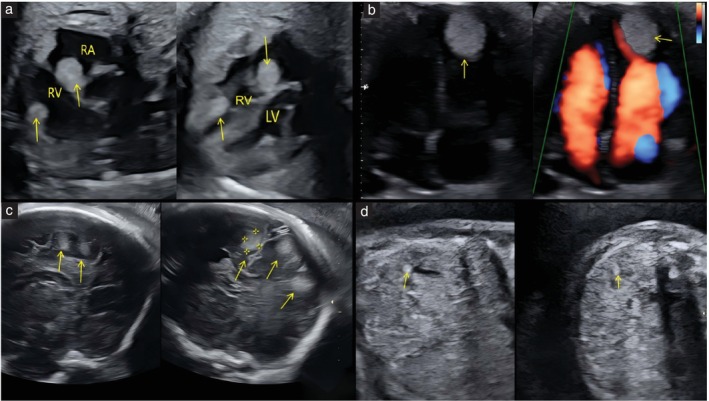
Fetal ultrasound examination at different gestational ages in typical cases of tuberous sclerosis complex. (a) Multiple nodular hyperechogenic masses (cardiac rhabdomyomas (CR)) (arrows), in the right atrium (RA) and right ventricle (RV) at 32 weeks' gestation. (b) A single nodular hyperechogenic mass (a CR) with no blood flow signal (arrow) in the left ventricular cavity at 29 weeks' gestation. (c) Multiple nodular hyperechogenic masses (arrows) within the intracranial brain parenchyma in a fetus presenting with multiple CR at 29 weeks' gestation. (d) A mass‐like patchy echo (arrow) in the renal region in a fetus presenting with multiple CR at 25 weeks' gestation. LV, left ventricle.

**Figure 3:**
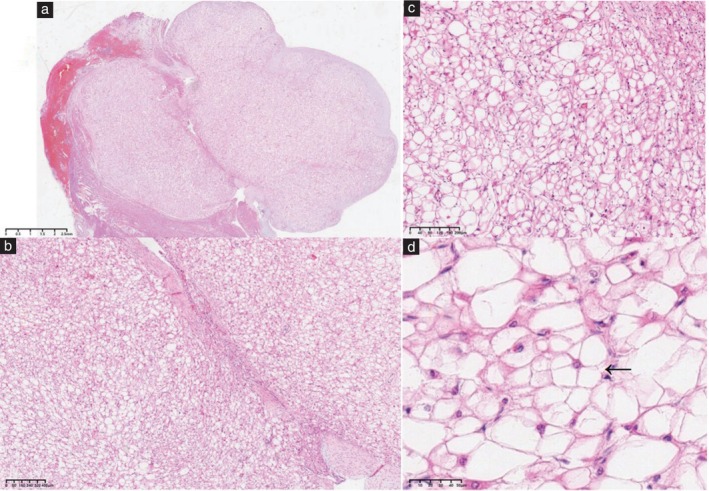
Histopathological features of cardiac rhabdomyoma: fetopsy specimen following termination of pregnancy. (a) Low‐power view (hematoxylin and eosin (H&E) stain; original magnification, ×10), showing a well‐circumscribed, lobulated mass with expansile nodular growth. (b) Intermediate view (H&E stain, ×40), revealing densely packed abnormal cardiomyocytes with prominent cytoplasmic clearing. (c) High‐power view (H&E stain, ×100), demonstrating cytoplasmic vacuolization and absence of fibrous stroma. (d) High‐power view (H&E stain, ×1000), highlighting the characteristic ‘spider cells’ (arrow), with uniformly eosinophilic, glycogen‐rich, vacuolated cytoplasm.

Between February 2016 and December 2024, 80 fetuses were diagnosed with CR at our center (Figure 1). Initial prenatal ultrasound screening identified CR in all included fetuses, presenting as either a single hyperechogenic mass or multiple hyperechogenic masses (Figure 2a,b); in a subset of TSC‐positive cases with classic multiorgan involvement, concurrent extracardiac manifestations, such as sonographically visible intracranial lesions (Figure 2c) or renal abnormalities (Figure 2d), were also detected at this baseline evaluation in fetuses presenting with multiple CR. Based on the integrated diagnostic criteria, 48 (60.0%) fetuses in our cohort were classified as TSC‐positive and 32 (40.0%) were classified as TSC‐negative. The definitive categorization of TSC was established through the multimodal integration of FE, brain MRI and trio‐WES (Table 1). Following this diagnostic sequence, 87.5% (*n* = 42) of the 48 TSC‐positive cases were diagnosed by imaging based on the prenatally adapted diagnostic criteria without the requirement for positive molecular findings. Notably, tumor multiplicity was strongly associated with diagnostic yield: among the TSC‐positive cases, trio‐WES served as the sole definitive diagnostic modality in 50.0% (3/6) of fetuses with a single CR, compared with only 7.1% (3/42) of those with multiple CR. Overall, diagnostic discordance was observed in nine cases within the TSC‐positive cohort: five cases were MRI‐negative but WES‐positive, whereas four were WES‐negative but were successfully diagnosed based on definitive MRI findings. All four cases in which trio‐WES was negative despite definitive MRI findings underwent TOP and in two of these cases the parents agreed to pathological examination. In both cases, the postmortem examination provided definitive validation of the prenatal imaging by demonstrating the characteristic ‘spider cells’ (Figure 3) specifically within the CR, alongside histopathologically confirmed characteristic cellular abnormalities in the intracranial lesions.

**Table 1:** Distribution of prenatal diagnostic pathways and pathological correlation in 48 fetuses with cardiac rhabdomyoma (CR) and confirmed tuberous sclerosis complex (TSC)

Diagnostic sequence			
CR multiplicity*	MRI	Trio‐WES	TSC‐positive
+	+	+	13 (27.1)
+	+	–	4 (8.3)
+	+	NP	22 (45.8)
+	–	+	2 (4.2)
+	NP	+	1 (2.1)
–	+	+	1 (2.1)
–	+	NP	2 (4.2)
–	–	+	3 (6.3)

Data are presented as *n* (%).

* Determined at ultrasound examination; note that, while the presence of a single CR was sufficient to fulfill the major clinical criterion, data are stratified by multiplicity to illustrate its impact on the diagnostic pathway. CR multiplicity +/–, multiple/single CR detected by fetal echocardiography; MRI +/–, positive/negative for major brain lesion identified by magnetic resonance imaging; NP, not performed; trio‐WES +/–, pathogenic/no pathogenic variants of *TSC1* or *TSC2* detected by trio whole‐exome sequencing.

Among the potential clinical predictors evaluated, tumor multiplicity and abnormal fetal brain MRI findings demonstrated a highly significant statistical association with a positive TSC diagnosis (both *P* < 0.001, Table 2). In contrast, baseline characteristics such as maternal age, gestational age at diagnosis and maximum tumor diameter showed no statistical association with TSC status. Tumors involving the right ventricle showed a trend towards higher prevalence in TSC‐positive fetuses (62.5% *vs* 40.6%, *P* = 0.069); other specific locations showed no significant association with TSC status (Table 2).

**Table 2:** Baseline prenatal characteristics, tumor multiplicity and location and fetal brain magnetic resonance imaging findings in 80 fetuses with cardiac rhabdomyoma, stratified by tuberous sclerosis complex (TSC) status

Characteristic	TSC‐positive (*n* = 48)	TSC‐negative (*n* = 32)	*P*
Maternal age (years)	30.0 (27.0–32.0)	30.0 (27.0–32.5)	> 0.9
GA at diagnosis (weeks)	26.5 (24.0–32.0)	29.0 (25.0–33.5)	0.3
Maximum tumor diameter (cm)	1.10 (0.80–1.25)	1.00 (0.80–1.30)	0.9
Tumor multiplicity			< 0.001
Multiple	42 (87.5)	16 (50.0)	
Single	6 (12.5)	16 (50.0)	
Tumor location			
Interventricular septum	23 (47.9)	10 (31.3)	0.2
Left ventricle	39 (81.3)	22 (68.8)	0.3
Right ventricle	30 (62.5)	13 (40.6)	0.069
Right atrium	5 (10.4)	3 (9.4)	> 0.9
Left atrium	1 (2.1)	1 (3.1)	> 0.9
Apex	1 (2.1)	0 (0)	> 0.9
Fetal brain MRI*			< 0.001
Negative	5/47 (10.6)	23/23 (100)	
Any major brain lesion	42/47 (89.4)	0/23 (0)	
SEN	25/47 (53.2)	0/23 (0)	
Cortical tuber	8/47 (17.0)	0/23 (0)	
SEN + cortical tuber	8/47 (17.0)	0/23 (0)	
SEGA	1/47 (2.1)	0/23 (0)	

Data are presented as median (interquartile range), *n* (%) or *n*/*N* (%).

* Magnetic resonance imaging (MRI) metrics available in 70 cases due to 10 patients declining to undergo further testing. GA, gestational age; SEGA, subependymal giant cell astrocytoma; SEN, subependymal nodule(s).

**Figure 4:**
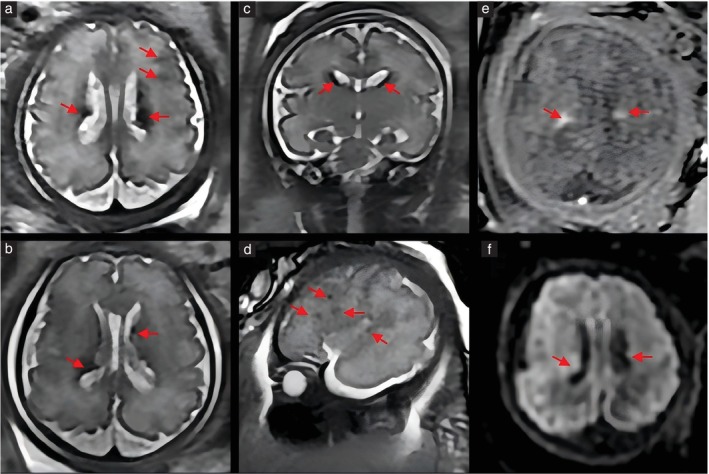
Prenatal brain findings on magnetic resonance imaging in cases of tuberous sclerosis complex. (a–d) Axial T2‐weighted images demonstrating multiple hypointense nodular lesions (arrows) with a characteristic distribution, including the subependymal region of the bilateral lateral ventricles, frontal white matter and left parietal white matter. (e) Axial T1‐weighted image, showing hyperintense subependymal nodules (arrows). (f) Axial diffusion‐weighted image revealing subependymal nodules with mild hyperintensity (arrows).

Table 3 presents diagnostic performance metrics. Both tumor multiplicity and fetal brain MRI demonstrated high sensitivity for TSC detection, but MRI showed far superior diagnostic utility, with 100.0% specificity and PPV, compared with the considerably lower specificity (50.0%) and PPV (72.4%) of tumor multiplicity. MRI successfully identified intracranial lesions, including SEN, cortical tubers and one case of SEGA, in 42/47 of the TSC‐positive cases that underwent MRI (Figure 4). Notably, 81.0% (34/42) of the intracranial lesions confirmed by MRI were occult on routine screening ultrasound examination. This establishes MRI as a decisive tool for clinical confirmation in this cohort.

**Table 3:** Diagnostic performance metrics of tumor multiplicity and fetal brain magnetic resonance imaging (MRI) in prediction of tuberous sclerosis complex (TSC) in fetuses with cardiac rhabdomyoma

Predictor	Sensitivity (%)	Specificity (%)	PPV (%)	NPV (%)
Tumor multiplicity	87.5 (74.8–95.3)	50.0 (31.9–68.1)	72.4 (59.1–83.3)	72.7 (49.8–89.3)
Fetal brain MRI*	87.5 (74.8–95.3)	100.0 (89.1–100.0)	100.0 (91.6–100.0)	84.2 (68.7–94.0)

Data in parentheses are 95% CI.

* MRI available in 70 cases (47 TSC‐positive and 23 TSC‐negative) due to 10 patients declining to undergo further testing. NPV, negative predictive value; PPV, positive predictive value.

### Genetic landscape and association between genotype and phenotype

Genetic analysis via trio‐WES was carried out in 31 cases and identified pathogenic or likely pathogenic variants in 20 of them (Table 4). Trio‐WES proved indispensable in diagnosing fetuses with atypical or occult imaging presentation, establishing a definitive diagnosis in five cases (two with multiple CR and three with a single CR) that presented with normal prenatal brain MRI. This underscores that, although MRI provided absolute specificity, it may lack the sensitivity to detect early‐stage or subtle CNS involvement in a minority of affected fetuses.

**Table 4:** Spectrum of 20 pathogenic and likely pathogenic genetic variants identified via trio whole‐exome sequencing in fetuses with cardiac rhabdomyoma (CR) that were tuberous sclerosis complex‐positive

Case	Gene	Variant (HGVS)	Predicted mutation	Inheritance	Imaging findings
1	*TSC1*	c.1525C>T:p (p.R509*)	Nonsense	*De novo*	Multiple CR, normal brain MRI
2	*TSC1*	c.1808‐c (p.P603Pfs*3)	Frameshift	*De novo*	Multiple CR, normal brain MRI
3	*TSC1*	c.1963C>T	Missense	*De novo*	Single CR, normal brain MRI
4	*TSC2*	c.370_372delTTT(p.Phe124del)	Protein alteration	Familial	Single CR, normal brain MRI
5	*TSC2*	c.824_826delACA(p.Asn275del)	Protein alteration	*De novo*	Multiple CR, multiple SEN
6	*TSC2*	c.1513C>T:p (p.R505*)	Nonsense	Familial	Multiple CR, multiple SEN
7	*TSC2*	c.1832G>A	Missense	*De novo*	Multiple CR, multiple cortical tubers
8	*TSC2*	c.2356‐1G>A	Missense	*De novo*	Multiple CR, multiple SEN
9	*TSC2*	c.2356‐2A>G	Missense	*De novo*	Single CR, multiple SEN and cortical tubers
10	*TSC2*	c.3412C>T	Missense	*De novo*	Multiple CR, multiple SEN and SEGA
11	*TSC2*	c.370_372delTTT (p.Phe124del)	In‐frame indel	Familial	Single CR, normal brain MRI
12	*TSC2*	c.4174C>T	Missense	Familial	Multiple CR, single SEN
13	*TSC2*	c.4289_4299delinsC (p.W1430Sfs*43)	Frameshift	Familial	Multiple CR, multiple SEN
14	*TSC2*	c.482‐2A>C	Missense	Familial	Multiple CR, multiple SEN
15	*TSC2*	c.4878delp (p.Thr1627ProfsTer4s)	Frameshift	*De novo*	Multiple CR, MRI data unavailable
16	*TSC2*	c.5160 + 2dupT	Splice site	*De novo*	Multiple CR, single cortical tuber
17	*TSC2*	c.5228G>A:p (p.R1743Q)	Missense	Familial	Multiple CR, multiple SEN
18	*TSC2*	c.5238_5255del (p.H1746)	In‐frame indel	Familial	Multiple CR, multiple SEN
19	*TSC2*	c.656T>C:p (p.L219P)	Missense	*De novo*	Multiple CR, single cortical tuber
20	*TSC2*	c.824_826delACA (p.Asn275del)	In‐frame indel	*De novo*	Multiple CR, multiple SEN

HGVS, Human Genome Variation Society nomenclature; MRI, magnetic resonance imaging; SEGA, subependymal giant cell astrocytoma(s); SEN, subependymal nodule(s).

There was a marked predominance of *TSC2* variants: these were identified in 85.0% (17/20) of cases, whereas *TSC1* variants occurred in only 15.0% (3/20) of cases. All three cases with a *TSC1* variant were associated with a relatively mild phenotype, presenting with no abnormal findings on fetal brain MRI (two with multiple CR and one with a single CR). In contrast, *TSC2* variants were mostly associated with more severe manifestations, including all cases involving cortical tubers or SEN, and the only identified case of SEGA.

### Factors influencing pregnancy outcome and clinical decision‐making

The final diagnosis and the severity of imaging findings were the primary determinants of pregnancy outcome. The TOP rate was significantly higher in the TSC‐positive group (87.5%) compared with the TSC‐negative group (62.5%, *P* < 0.01). Statistical analysis confirmed the presence of multiple tumors (*P* < 0.001) and abnormal fetal brain MRI findings (*P* = 0.013) as key factors driving parental decision‐making regarding TOP (Table 5). This high rate of TOP in more severe cases created a notable selection bias in the subsequent analysis of liveborn infants.

**Table 5:** Comparison of prenatal features potentially influencing parental decision‐making regarding termination of pregnancy (TOP) in 80 fetuses with cardiac rhabdomyoma

Prenatal feature	Live birth (*n* = 18)	TOP (*n* = 62)	*P*
Maternal age (years)	29.5 (27.0–32.0)	30.0 (27.0–33.0)	0.5
GA at diagnosis (weeks)	30.0 (26.0–35.0)	26.5 (24.0–32.0)	0.054
Maximum tumor diameter (cm)	0.95 (0.7–1.2)	1.05 (0.8–1.3)	0.8
Tumor multiplicity			< 0.001
Multiple	6 (33.3)	52 (83.9)	
Single	12 (66.7)	10 (16.1)	
Fetal brain MRI findings*			0.013
Negative	10/14 (71.4)	18/56 (32.1)	
Positive	4/14 (28.6)	38/56 (67.9)	
Final TSC diagnosis			0.013
TSC‐positive	6 (33.3)	42 (68.0)	
TSC‐negative	12 (66.7)	20 (32.0)	

Data are presented as median (interquartile range), *n* (%) or *n*/*N* (%).

* Magnetic resonance imaging (MRI) metrics available in 70 cases due to 10 patients declining to undergo further testing. GA, gestational age; TSC, tuberous sclerosis complex.

### Pregnancy neurological outcomes and long‐term follow‐up

Follow‐up data were available for all 18 liveborn infants (Table 6). Regarding perinatal metrics, although 1‐min Apgar scores were similar between the groups, birth weight demonstrated a statistically significant difference, with TSC‐positive neonates having lower birth weights (*P* = 0.025). Most notably, neurological outcomes were divergent: 100% (6/6) of TSC‐positive live births developed postnatal epilepsy, compared with none (0/12) in the TSC‐negative group (*P* < 0.001). Furthermore, spontaneous CR regression was observed in 50.0% (6/12) of the TSC‐negative infants, whereas no (0/6) tumor regression occurred in the TSC‐positive group (*P* = 0.054).

**Table 6:** Perinatal outcomes and postnatal neurological status of 18 liveborn infants that had fetal cardiac rhabdomyoma, stratified by tuberous sclerosis complex (TSC) status

Characteristic	TSC‐positive (*n* = 6)	TSC‐negative (*n* = 12)	*P*
Gestational age at delivery			0.6
38 weeks	1 (16.7)	3 (25.0)	
39 weeks	0 (0)	2 (16.7)	
40 weeks	5 (83.3)	6 (50.0)	
41 weeks	0 (0)	1 (8.3)	
Birth weight (g)	3050 (2730–3230)	3500 (3230–3740)	0.025
1‐min Apgar score of 10*	4/4 (100)	12 (100)	1.0
Spontaneous postnatal tumor regression	0 (0)	6 (50.0)	0.054
Postnatal epilepsy	6 (100)	0 (0)	< 0.001

Data are presented as *n* (%), median (interquartile range) or *n*/*N* (%).

* Two cases did not have data for 1‐min Apgar score in the liveborn TSC‐positive cohort.

### Diagnostic nuances of single cardiac rhabdomyomas

To address the possibility that single cardiac tumors may represent pathologies other than CR (such as fibromas or teratomas), we conducted a verification analysis of the 22 cases with a single tumor (of which 12 were liveborn and 10 underwent TOP) (Table S1). The diagnosis of CR was confirmed through pathological examination in one case (identifying the characteristic spider cells) and through syndromic association in six TSC‐positive cases. Among the 12 that were TSC‐negative and liveborn, spontaneous regression (a hallmark feature unique to CR) was observed in two cases during postnatal follow‐up. The remaining cases exhibited typical hyperechogenic imaging characteristics and a benign, non‐progressive clinical course, which was inconsistent with the aggressive growth or complex morphology of cardiac fibromas or teratomas. This collective evidence supports our classification of these single tumors as CR.

## DISCUSSION

The prenatal identification of CR serves as a pivotal diagnostic opportunity for TSC. In this longitudinal cohort of 80 fetuses, we have demonstrated that although tumor multiplicity is a sensitive screening indicator, the integrated application of fetal brain MRI and trio‐WES is necessary to achieve diagnostic specificity and inform prognosis. Furthermore, trio‐WES extends beyond establishing a definitive diagnosis by enabling the differentiation of *TSC1* and *TSC2* variants; this molecular stratification provides essential prognostic insight, as the specific mutated genes appear to be closely associated with the severity of the fetal phenotype.

### Diagnostic performance and the role of fetal MRI

In our cohort, tumor multiplicity achieved a sensitivity of 87.5% for TSC, yet its low specificity (50.0%), with wide 95% CI (31.9–68.1%), highlights significant diagnostic overlap with non‐TSC fetuses^6^, ^17^. This diagnostic limitation of sonography was effectively addressed by fetal brain MRI, which demonstrated a specificity and positive predictive value of 100.0% in our cohort.

Notably, 81.0% of the intracranial lesions identified by fetal brain MRI were occult on routine screening ultrasound examinations. These findings align with previous clinical reports highlighting the utility of prenatal MRI for detecting major CNS defects, particularly SEN and cortical tubers^18^. Specifically, previous cohort data stated that MRI enabled definitive prenatal TSC diagnosis by identifying SEN in 100.0% and cortical tubers in 61.5% of affected cases, often far earlier than the typical postnatal onset of epilepsy^10^. Our study reinforces that, while tumor multiplicity is a sensitive screening tool, achieving diagnostic specificity required integration of fetal brain MRI and trio‐WES: MRI detected structural lesions in four WES‐negative cases, and WES secured a diagnosis in five fetuses with normal brain imaging. The updated 2021 International TSC Consensus criteria for diagnosis of TSC rely heavily on clinical features that manifest only postnatally^1^, ^15^. By adapting them to enable use of exclusively prenatally observable major features, namely CR on FE and intracranial lesions on MRI, we established a rigorous prenatal diagnostic framework that included molecular findings or the combination of CR and MRI‐detected CNS lesions.

### Characterization of diagnostic discordance and technical limitations

This study elucidates some biological and technical reasons for imaging–genomic discordance in fetal TSC. Even with a normal fetal brain MRI result, there remains a substantial residual risk that a fetus harbors TSC, which may manifest only postnatally, due to the developmental timing of CNS lesions^1^, ^12^. In our study, trio‐WES‐positive/MRI‐negative cases accounted for 10.4% of the TSC‐positive cohort, primarily due to the chronological maturation of the fetal brain, because TSC‐associated hamartomas are developmental and may remain below the detection threshold before 24 weeks' gestation^9^, ^10^, ^12^. Conversely, trio‐WES‐negative/MRI‐positive cases comprised 8.3% of the TSC‐positive cohort, likely due to the presence of deeply intronic variants being missed by WES or low‐level somatic mosaicism^19^, ^20^, ^21^. These technical nuances underscore that fetal MRI and trio‐WES are best used as concurrent and complementary tests rather than as alternative tests; a negative result from either modality is insufficient to rule out TSC in high‐risk fetuses.

### Genomic landscape and association between genotype and phenotype

The mutation spectrum in our cohort was heavily weighted toward *TSC2* variants (85.0%); this exceeds the typical distribution seen in general postnatal TSC populations, in which *TSC2* variants generally account for 60–70% of identified cases^22^, ^23^. This enrichment likely reflects the more aggressive fetal tumor burden associated with *TSC2* variants, rendering these cases more detectable during routine second‐trimester screening^24^. Consistent with previous research, fetuses with *TSC2* variants in this study presented with more severe manifestations^23^. This structural severity aligns with the established literature, which demonstrates that *TSC2* variants frequently result in severely truncated tuberin proteins, a mechanism known to drive high overall neurological morbidity^25^. In contrast, *TSC1* variants were associated with milder phenotypes in our cohort, with all three cases having normal fetal brain MRI. This supports recent literature suggesting that *TSC1* variants frequently manifest with a milder fetal phenotype that may evade detection during the second trimester^22^, ^24^, ^26^. Furthermore, tumors involving the right ventricle showed a trend toward higher prevalence in TSC‐positive fetuses (62.5% *vs* 40.6%, *P* = 0.069), suggesting that right ventricular involvement may serve as a subtle clinical clue for syndromic association.

### Clinical management of single cardiac rhabdomyomas and perinatal outcomes

The management of single CR is challenging; 27.3% (6/22) of cases with a single CR in this cohort were ultimately confirmed as TSC‐positive. Fetuses with single CR detected at ultrasound examination present a distinct diagnostic challenge, as they are even less likely to exhibit concurrent MRI‐detectable brain lesions compared to fetuses with multiple CRs. Consequently, these cases rely disproportionately on trio‐WES, underscoring the absolute necessity of molecular testing when imaging alone fails to meet clinical diagnostic criteria. Regarding pregnancy outcomes, the TOP rate in our cohort was significantly higher in the TSC‐positive group (87.5%). However, the TOP rate was high (62.5%) even in the TSC‐negative group, reflecting profound parental anxiety and the challenges involved in prenatal counseling for fetal CR. Postnatal follow‐up revealed that 100% (6/6) of the liveborn TSC‐positive infants developed epilepsy, which is a higher rate than that reported in the general postnatal TSC population (70–90%)^7^, ^27^. This likely reflects a selection bias in our study cohort whereby families with less severe imaging findings were more likely to continue the pregnancy.

Notably, TSC‐positive infants had significantly lower birth weights compared with TSC‐negative infants (median, 3050 g *vs* 3500 g; *P* = 0.025). This may suggest subtle intrauterine growth restriction or reflect more proactive medical intervention leading to earlier planned deliveries in the high‐risk cohort. Currently, the primary value of prenatal diagnosis is to facilitate proactive postnatal care, including immediate neonatal electroencephalogram (EEG) monitoring and pre‐emptive vigabatrin treatment, which can improve long‐term neurodevelopmental trajectories^10^, ^28^, ^29^. Evidence suggests that pre‐emptive treatment with vigabatrin upon detection of these subclinical abnormalities significantly reduces the risk of drug‐resistant epilepsy and improves long‐term neurodevelopmental outcome^30^, ^31^. Transplacental mTOR inhibitors were not used in this cohort due to the absence of life‐threatening hemodynamic compromise, but they represent a promising intervention for severe cases^17^, ^32^, ^33^. Perinatal management for liveborn infants in this cohort necessitated standard antiepileptic therapy; the initiation of mTOR inhibitors was not indicated, as these are generally reserved for cases involving refractory seizures, enlarging SEGA or progressive renal lesions^34^, ^35^, ^36^.

### Limitations

The single‐center, retrospective design of this study may have introduced selection bias. Diagnostic data were not uniformly available for all fetuses, and standard trio‐WES may miss extremely low‐level mosaicism. Furthermore, although we observed a distinct association between *TSC1* variants and milder prenatal phenotypes, the number of *TSC1* cases in our cohort was small (*n* = 3). Larger multicenter studies are necessary to draw definitive conclusions regarding the consistency of prenatal genotype–phenotype associations in TSC. The most significant limitation is the profound selection bias stemming from the 87.5% TOP rate in the TSC‐positive group, which was influenced directly by the diagnostic findings under investigation.

### Conclusion

An integrated multimodal diagnostic approach is essential for the accurate prenatal assessment of fetuses with CR. Although the detection of multiple CR on screening ultrasound examination and a major lesion on fetal brain MRI are key prenatal predictors of TSC, trio‐WES serves as a critical complementary tool, particularly for identifying etiology in early gestation, when brain lesions may not yet be detectable, and for assessing future recurrence risk. Our study highlights a significant association between genotype and phenotype, with *TSC2* variants driving more aggressive fetal phenotypes and higher neurological morbidity. Furthermore, the observation of lower birth weights in TSC‐positive infants underscores the profound biological impact of this syndromic condition on fetal development, while the high rate of TOP observed even among TSC‐negative cases highlights the extreme parental anxiety and complex decision‐making challenges triggered by the initial prenatal detection of CR. Ultimately, the high risk of intractable epilepsy in liveborn TSC infants necessitates a shift towards proactive perinatal care. Establishing a definitive prenatal diagnosis allows for immediate neonatal EEG monitoring and pre‐emptive intervention, which are vital for improving long‐term neurodevelopmental outcomes in this complex patient population.

## Supporting information


**Table S1** Diagnostic subanalysis and logic for verification of cardiac rhabdomyoma (CR) in 22 fetuses initially presenting with a single CR.

## Data Availability

The data that support the findings of this study are available on request from the corresponding author. The data are not publicly available due to privacy or ethical restrictions.

## References

[1] Northrup H , Aronow ME , Bebin EM , et al. Updated international tuberous sclerosis complex diagnostic criteria and surveillance and management recommendations. Pediatr Neurol. 2021;123:50‐66. 34399110 10.1016/j.pediatrneurol.2021.07.011

[2] Crino PB , Nathanson KL , Henske EP . The tuberous sclerosis complex. N Engl J Med. 2006;355(13):1345‐1356. 17005952 10.1056/NEJMra055323

[3] Curatolo P , Moavero R , De Vries PJ . Neurological and neuropsychiatric aspects of tuberous sclerosis complex. Lancet Neurol. 2015;14(7):733‐745. 26067126 10.1016/S1474-4422(15)00069-1

[4] Isaacs H . Fetal and neonatal cardiac tumors. Pediatr Cardiol. 2004;25(3):252‐273. 15360117 10.1007/s00246-003-0590-4

[5] Zhai S , Liu L , Yuan L , et al. Study on prenatal diagnosis and pregnancy outcome analysis of fetuses with cardiac rhabdomyoma. J Perinatol. 2023;43(7):864‐870. 37330616 10.1038/s41372-023-01673-w

[6] Milon V , Malinge MC , Blanluet M , et al. Diagnosis of tuberous sclerosis in the prenatal period: a retrospective study of 240 cases and review of the literature. Eur J Hum Genet. 2024;32(12):1590‐1598. 38806662 10.1038/s41431-024-01631-w PMC11606953

[7] Ding Y , Wang J , Zhou Y , et al. Quality of life in children with tuberous sclerosis complex: a pediatric cohort study. CNS Neurosci Ther. 2021;27(3):280‐288. 33225634 10.1111/cns.13473 PMC7871787

[8] Qi Y , Ding H , Huang Y , et al. A multidisciplinary approach in prenatal diagnosis of TSC with cardiac rhabdomyoma as the initial symptom. Front Pediatr. 2021;9:628238. 34513752 10.3389/fped.2021.628238 PMC8429840

[9] Goergen SK , Fahey MC . Prenatal MR imaging phenotype of fetuses with tuberous sclerosis: an institutional case series and literature review. AJNR Am J Neuroradiol. 2022;43(4):633‐638. 35332020 10.3174/ajnr.A7455 PMC8993194

[10] Bekiesinska‐Figatowska M , Sobieraj P , Pasieczna M , Szymkiewicz‐Dangel J . Early diagnosis of tuberous sclerosis complex: prenatal diagnosis. Am J Neuroradiol. 2023;44(9):1070‐1076. 37536734 10.3174/ajnr.A7952 PMC10494953

[11] Zeng Z , Zhang L , Zhou Y , et al. Clinical utility of trio whole exome sequencing in fetuses with ultrasound anomalies. Hum Genomics. 2025;19(1):37. 40188065 10.1186/s40246-025-00745-6 PMC11972492

[12] Hulshof HM , Slot EMH , Lequin M , et al. Fetal brain magnetic resonance imaging findings predict neurodevelopment in children with tuberous sclerosis complex. J Pediatr. 2021;233:156‐162.e2. 33640330 10.1016/j.jpeds.2021.02.060

[13] Ge Y , Yuan H , Yu Y , et al. Application of family whole‐exome sequencing for prenatal diagnosis‐an analysis of 357 cases. Front Med. 2025;12:1529894. 10.3389/fmed.2025.1529894 PMC12360300 40832109

[14] Tyburczy ME , Dies KA , Glass J , et al. Mosaic and intronic mutations in TSC1/TSC2 explain the majority of TSC patients with no mutation identified by conventional testing. PLOS Genet. 2015;11(11):e1005637. 26540169 10.1371/journal.pgen.1005637 PMC4634999

[15] Northrup H , Krueger DA , International Tuberous Sclerosis Complex Consensus Group . Tuberous sclerosis complex diagnostic criteria update: recommendations of the 2012 International Tuberous Sclerosis Complex Consensus Conference. Pediatr Neurol. 2013;49(4):243‐254. 24053982 10.1016/j.pediatrneurol.2013.08.001 PMC4080684

[16] Richards S , Aziz N , Bale S , et al. Standards and guidelines for the interpretation of sequence variants: a joint consensus recommendation of the American College of Medical Genetics and Genomics and the Association for Molecular Pathology. Genet Med. 2015;17(5):405‐424. 25741868 10.1038/gim.2015.30 PMC4544753

[17] Mustafa HJ , Javinani A , Morning ML , et al. Characteristics and outcomes of fetal cardiac rhabdomyoma with or without mTOR inhibitors, a systematic review and meta‐analysis. Prenat Diagn. 2024;44(10):1251‐1267. 39164800 10.1002/pd.6640

[18] Gao J , Wu Y , Ma D , et al. Genetic results and clinical outcome of fetuses with cardiac rhabdomyoma including a novel mutation in TSC2 gene. Clin Chim Acta. 2025;577:120433. 40532806 10.1016/j.cca.2025.120433

[19] Ye Z , Lin S , Zhao X , et al. Mosaicism in tuberous sclerosis complex: lowering the threshold for clinical reporting. Hum Mutat. 2022;43(12):1956‐1969. 36030538 10.1002/humu.24454

[20] Ambrosetti I , Cristalli CP , Montanari F , et al. Low‐Level Mosaicism in Tuberous Sclerosis Complex (TSC): diagnostic and clinical implications from two novel cases and literature review. Am J Med Genet A. 2026;200(4):862‐870. 41486106 10.1002/ajmga.70018

[21] Manzanilla‐Romero HH , Weis D , Schnaiter S , Rudnik‐Schöneborn S . Low‐level mosaicism in tuberous sclerosis complex in four unrelated patients: comparison of clinical characteristics and diagnostic pathways. Am J Med Genet A. 2021;185(12):3851‐3858. 34328706 10.1002/ajmg.a.62433 PMC9291125

[22] Dabora SL , Jozwiak S , Franz DN , et al. Mutational analysis in a cohort of 224 tuberous sclerosis patients indicates increased severity of TSC2, compared with TSC1, disease in multiple organs. Am J Hum Genet. 2001;68(1):64‐80. 11112665 10.1086/316951 PMC1234935

[23] Reyna‐Fabián ME , Hernández‐Martínez NL , Alcántara‐Ortigoza MA , et al. First comprehensive TSC1/TSC2 mutational analysis in Mexican patients with Tuberous Sclerosis Complex reveals numerous novel pathogenic variants. Sci Rep. 2020;10(1):6589. 32313033 10.1038/s41598-020-62759-5 PMC7170856

[24] Jesmanas S , Norvainytė K , Gleiznienė R , Šimoliūnienė R , Endzinienė M . Different MRI‐defined tuber types in tuberous sclerosis complex: quantitative evaluation and association with disease manifestations. Brain Dev. 2018;40(3):196‐204. 29258718 10.1016/j.braindev.2017.11.010

[25] Hodges AK . Pathological mutations in TSC1 and TSC2 disrupt the interaction between hamartin and tuberin. Hum Mol Genet. 2001;10(25): 2899‐2905. 11741833 10.1093/hmg/10.25.2899

[26] Malinger G , Prabhu A , Maroto González A , et al. Fetal neurosonography as accurate tool for diagnosis of brain involvement in tuberous sclerosis. Ultrasound Obstet Gynecol. 2023;62(3):391‐397. 37021742 10.1002/uog.26213

[27] McDonald NM , Hyde C , Choi AB , et al. Improving developmental abilities in infants with tuberous sclerosis complex: a pilot behavioral intervention study. Infants Young Child. 2020;33(2):108‐118. 32467653 10.1097/iyc.0000000000000160 PMC7255424

[28] Jóźwiak S , Kotulska K , Domańska‐Pakieła D , et al. Antiepileptic treatment before the onset of seizures reduces epilepsy severity and risk of mental retardation in infants with tuberous sclerosis complex. Eur J Paediatr Neurol. 2011;15(5): 424‐431. 21507691 10.1016/j.ejpn.2011.03.010

[29] Krueger DA , Northrup H , Northrup H , et al. Tuberous sclerosis complex surveillance and management: recommendations of the 2012 international tuberous sclerosis complex consensus conference. Pediatr Neurol. 2013;49(4):255‐265. 24053983 10.1016/j.pediatrneurol.2013.08.002 PMC4058297

[30] Jozwiak S , Słowińska M , Borkowska J , et al. Preventive antiepileptic treatment in tuberous sclerosis complex: a long‐term, prospective trial. Pediatr Neurol. 2019;101:18‐25. 31481332 10.1016/j.pediatrneurol.2019.07.008

[31] Wang X , Ding Y , Zhou Y , et al. Prenatal diagnosis and intervention improve developmental outcomes and epilepsy prognosis in children with tuberous sclerosis complex. Dev Med Child Neurol. 2022;64(10):1230‐1236. 35612999 10.1111/dmcn.15265

[32] Martinez‐Garcia A , Tirado‐Aguilar OA , Acevedo‐Gallegos S , et al. Fetal cardiac rhabdomyomas susceptible to prenatal treatment with mTOR inhibitors: literature review and proposal of a prenatal management algorithm. Front Med. 2025;12:1711774. 10.3389/fmed.2025.1711774 PMC12719440 41438139

[33] Uno K , Nomura Y , Kawaguchi M , et al. Transplacental sirolimus: a new treatment strategy for life‐threatening fetal cardiac rhabdomyomas—a case report. Orphanet J Rare Dis. 2025;20(1):291. 40490815 10.1186/s13023-025-03780-7 PMC12147371

[34] Krueger DA , Wilfong AA , Mays M , et al. Long‐term treatment of epilepsy with everolimus in tuberous sclerosis. Neurology. 2016;87(23):2408‐2415. 27815402 10.1212/WNL.0000000000003400 PMC5177677

[35] Mongrain V , van Doesburg NH , Rypens F , et al. A case report of severe tuberous sclerosis complex detected in utero and linked to a novel duplication in the TSC2 gene. BMC Neurol. 2020;20(1):324. 32873234 10.1186/s12883-020-01905-y PMC7460776

[36] Karagianni A , Karydakis P , Giakoumettis D , Nikas I , Sfakianos G , Themistocleous M . Fetal subependymal giant cell astrocytoma: a case report and review of the literature. Surg Neurol Int. 2020;11:26. 32123614 10.25259/SNI_10_2019 PMC7049878

