# Supplementary material for: Prenatal diagnosis of cardiac rhabdomyoma: implications for predicting tuberous sclerosis complex and guiding perinatal management
Source: Ultrasound Obstet Gynecol. 2026 Jun 6;68(1):88–98. doi: 10.1002/uog.70240 (PMC13325691; doi:10.1002/uog.70240)
Supplement: Supplementary file 1 — Table S1 Diagnostic subanalysis and logic for verification of cardiac rhabdomyoma (CR) in 22 fetuses initially presenting with a single CR. [file UOG-68-88-s001.docx]

**Table S1** Diagnostic subanalysis and logic for verification of cardiac rhabdomyoma (CR) in 22 fetuses initially presenting with a single CR

| Method of verification | *n* (%) | Pregnancy outcome  (live birth/TOP (*n*)) | Clinical reasoning and evidence |
| --- | --- | --- | --- |
| Pathological confirmation (fetopsy) | 1 (4.5) | 0/1 | Confirmed via autopsy to have a single CR |
| Definitive clinical confirmation (TSC-positive) | 6 (27.3) | 2/4 | Syndromic association: in context of TSC (confirmed via MRI/Trio-WES), cardiac tumors are histologically confirmed as CR in > 95% cases |
| Postnatal spontaneous regression | 2 (9.1) | 2/0 | Characteristic behavior: tumors regressed during follow-up; spontaneous regression is a pathognomonic feature of CR, not seen in fibromas or teratomas |
| Typical US features and stable course | 13 (59.1) | 8/5 | Imaging logic: all cases presented as well-defined, homogeneous, hyperechogenic masses within the ventricles/myocardium, lacking the calcification of fibromas and the cystic components of teratomas; liveborn cases remained stable without growth |

## WES, whole-exome sequencing; MRI, magnetic resonance imaging; TSC, tuberous sclerosis complex; US, ultrasound.
